# A Brief Overview of Molecular Biology in Pituitary Adenomas with a Focus on Aggressive Lesions

**DOI:** 10.3390/ijms26083717

**Published:** 2025-04-15

**Authors:** Ligia Gabriela Tataranu

**Affiliations:** 1 Department of Neurosurgery, Carol Davila University of Medicine and Pharmacy, 020021 Bucharest, Romania; ligia.tataranu@umfcd.ro; 2 Department of Neurosurgery, Bagdasar-Arseni Emergency Clinical Hospital, 041915 Bucharest, Romania

**Keywords:** pituitary neuroendocrine tumors, pituitary adenomas, cellular and molecular biology, molecular pathology, hypophysis

## Abstract

Arising from the anterior lobe of the hypophysis, pituitary neuroendocrine tumors (PitNETs), previously known as pituitary adenomas, constitute an intricate and heterogeneous entity. Although they are defined as benign pathology, these tumors can often invade neighboring structures and demonstrate aggressive behavior. The continuous advancement of molecular biology has begun to shed light on the genetic mutations behind the development and evolution of this pathology, providing a better understanding. Notwithstanding, the rising occurrence in recent decades can mainly be attributed to advanced diagnostic techniques; however, these tumors continue to increase in prevalence and incidence, creating a heavy burden on healthcare systems. Consequently, the need for further studies is dire, primarily due to a lack of tailored therapeutic approaches. Thus, this article aims to offer a brief overview of the molecular biology behind these complex tumors to contribute, even on a small scale, to more comprehensive care.

## 1. Introduction

About 90% of tumors originating from the pituitary gland are adenomas, which occur more frequently in females. Additionally, pituitary adenomas (PAs) account for up to 15% of all intracranial tumors found in adults [1,2]. The incidence of these tumors has increased in recent decades, mainly due to advanced diagnostic techniques, creating a heavy burden on healthcare systems, as well as on patients [3]. In the United States, the incidence is reported to be 4.20 per 100.000 population, with a predominance of African Americans [2]. Furthermore, in Europe, Canada, and Argentina, the trend toward increasing incidence and prevalence is still significant, with a reported overall yearly incidence of 5.1 per 100.000 and a mean prevalence of 89.1 per 100.000 with a female predominance [4].

In regard to the nomenclature changes, the fifth edition of the World Health Organization (WHO) Classification of Endocrine and Neuroendocrine Tumors categorizes adenohypophyseal tumors as (1) pituitary neuroendocrine tumors (PitNETs), which were previously known as pituitary adenomas; (2) pituitary blastoma; and (3) different types of craniopharyngioma [5]. Moreover, this classification identifies lesions based on cell lineage as determined by the expression of transcription factors, hormones, and biomarkers [5]. Although the classification has been generally accepted, there are still existing controversies among different authors. It is worth mentioning that initially, the International Pituitary Pathology Club suggested the term PitNET specifically for pituitary adenomas with aggressive behavior, mentioning that the term adenoma itself would not be appropriate to describe an aggressive and invasive tumor with highly proliferative status and persistent hypersecretory syndrome [6]. However, since the name change does not impact therapeutic management currently, many authors have decided not to apply this change, citing a lack of theoretical support, more significant confusion, and additional patient concerns and anxiety [7,8,9].

In 2024, Ho et al. proposed a new clinical classification that not only includes non-resected tumors but also provides an opportunity to evaluate the severity of the disease and the prognosis and assess the therapeutic response. The authors suggest that the last classification, from 2022, does not impact therapeutic management and can only be applied to resected tumors [10]. The classification evaluates nine factors, which are represented by the secretory status of the tumor, histopathologic result, phenotype, hypopituitarism, tumoral volume, mass effect, invasiveness, tumoral remnants, and genetic syndromes. Each of these factors is assigned a score of 0 (absence) or 1 (presence), while a score of 2 is specifically associated with factors correlated to an unfavorable prognosis. The authors consider that this proposed classification will better assess the overall risk and could transform management and research for each individual with PAs, despite a few limitations [10].

Nevertheless, new proposed classifications are yet to be evaluated and discussed in the coming years.

## 2. A Brief Overview of Molecular Biology

**Figure 1:**
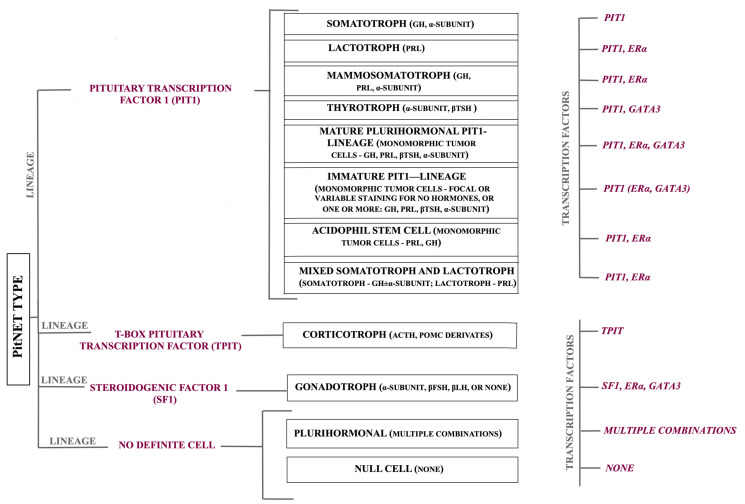
A summary of PitNET types and their transcription factors; ERα—estrogen receptor alpha; GATA3—Erythroid transcription factor 3; GH—growth hormone; PRL—prolactin; βTSH—beta-thyroid-stimulating hormone; ACTH—adrenocorticotropic hormone; POMC—proopiomelanocortin; βFSH—follicle-stimulating hormone; βLH—luteinizing hormone. The image highlights not only specific cellular types and subtypes but also PAs with unusual cell differentiation. Unlike in the previous classification, mammosomatotroph tumors are now a distinct relevant entity, and along with somatotroph, lactotroph, and thyrotroph tumors, they are of PIT1 lineage. Nevertheless, corticotroph tumors are of TPIT lineage, while gonadotroph tumors are of SF1 lineage. The parentheses exposed the hormones specific to each tumor, while it is worth mentioning that mixed tumors comprise two distinct cell types, both morphologically and immunohistochemically, and their ERα transcription factor is positive in the lactotroph tumor component. Although the majority of PAs have subtypes, thyrotroph and gonadotroph tumors do not have any. The presented figure also puts into the spotlight a new element represented by pitNETs considered tumors of precursor cells, such as acidophil stem cell and immature PIT1-lineage tumors, that are frequently plurihormonal, with very few exceptions. Furthermore, the figure exposes another new entity named mature plurihormonal PIT1-lineage tumor that is similar to mammosomatotrophic tumors but secretes supplementary hormones besides GH and PRL.

It is well known that PAs are distinguished as functional and non-functional based on their secretory activity if the hormone secretion is not detected in the blood or does not cause any clinical manifestation [11]. However, although these tumors are now categorized based on transcription factors and hormone production, the pituitary gland is a very complex entity, and it can be challenging or even unfeasible to use one significant pattern of molecules as biomarkers to elucidate different molecular mechanisms or to predict a prognosis and a therapeutic response [12]. The latest classification of pituitary tumors proposes a more comprehensive approach while highlighting detailed pathological characteristics [5] (Figure 1).

Regarding pituitary tumorigenesis, a succession of molecular alterations has been described in recent decades. Results based on proteomic analysis showed abnormal mitochondrial functioning, as well as oxidative dysregulation, and MAPK signaling dysfunctions [13]. Besides the proteomic alterations, peptidomic, transcriptomic, metabolomic, and genomic variations have been described [14]. Furthermore, biomarkers from the radiome can be used to create an integrative model that will better identify patients with PAs [15]. These “omics” are known as modern multi-omics and are connected and work through a network system that represents the central role in the present molecular pituitary approach [12].

Many of the molecules involved in the development and evolution of PAs are related to each other, which might indicate that this disease results from the accumulative consequences of genetic and epigenetic modifications [16].

Approximately 35% of all PAs are invasive into the surrounding anatomical structures: the cavernous sinus (evaluated through Knosp Grade), the sinus mucosa, paranasal sinuses, the upper parenchymal brain layer, the optic nerve, and the hypothalamus [17,18]. In these cases, gross total resection may be challenging and not always feasible because of the complex anatomical neighboring structures, and the risk of recurrence increases [17]. Out of all invasive PAs, approximately 10% of them have additional features such as a rapid development and recurrence rate, and they are refractory to standard therapies. These tumors are considered aggressive [19].

**Figure 2:**
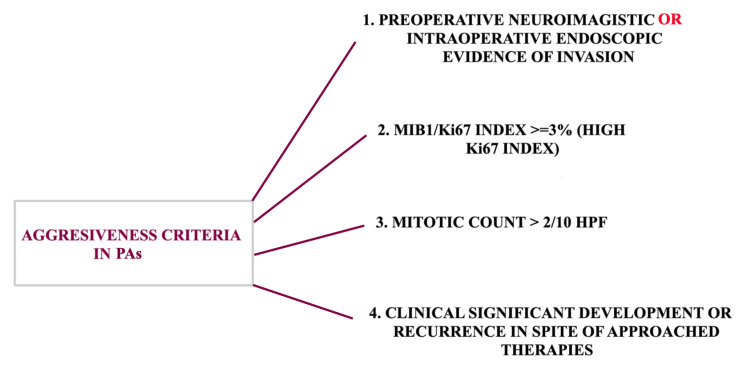
Specific features of aggressiveness in PAs. Tumoral infiltration can be confirmed only by neuroimaging results, while intraoperative evidence is additional. The Ki67 index and tumoral growth are not necessarily required criteria for diagnosing aggressive PAs, although they are still assessed. In a likewise manner, the recurrence rate is not mandatory in order to diagnose aggressive PAs.

Although it has been concluded that an aggressive tumor is always invasive, an invasive tumor is not necessarily aggressive. However, the invasiveness of the PAs can predict tumoral recurrence, given that in these cases, a more intense biomolecular activity was observed compared to that of non-invasive tumors [20]. The European Society of Endocrinology (ESE) recommends the use of the term “aggressive” for tumors that are radiologically or surgically invasive and have a rapid progression despite conventional treatments [21]. Furthermore, the European Pituitary Pathology Group defined precise features to diagnose aggressive behavior (Figure 2).

**Figure 3:**
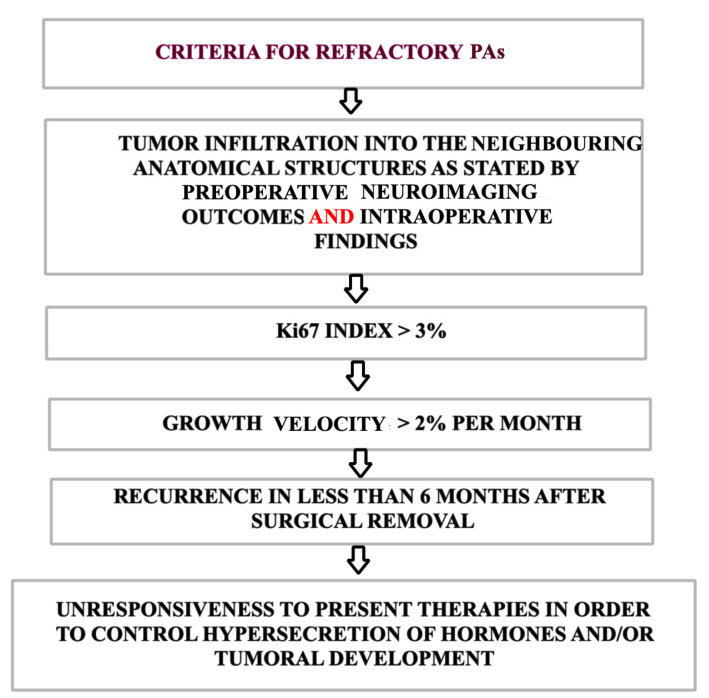
Specific characteristics of refractory PAs. Unlike aggressive PAs, the tumoral infiltration of refractory tumors must be confirmed both by neuroimaging results and intraoperative results. Moreover, the Ki67 index, growth velocity, and recurrences are more strict criteria when diagnosing refractory PAs, while resistance to standard therapies is similar in both.

Dai et al. proposed the term “refractory” to identify an invasive tumor with an increased Ki-67 index, an unusually rapid growth rate, early recurrence, and resistance to standard therapies (Figure 3). The authors considered that this term would better express aggressiveness in PAs while having additional advantages, such as eliminating the confusion regarding radiological versus intraoperative invasion, which sometimes can differ [17].

Notwithstanding how the definitions of aggressive PAs and refractory PAs may overlap, some characteristics are more strict for refractoriness, and this term was proposed in order to have more accurate and objective diagnosis criteria [22]. Thus, we summarized the different characteristics between the two entities in Table 1.

### 2.1. Inherited Pituitary Tumors

**Figure 4:**
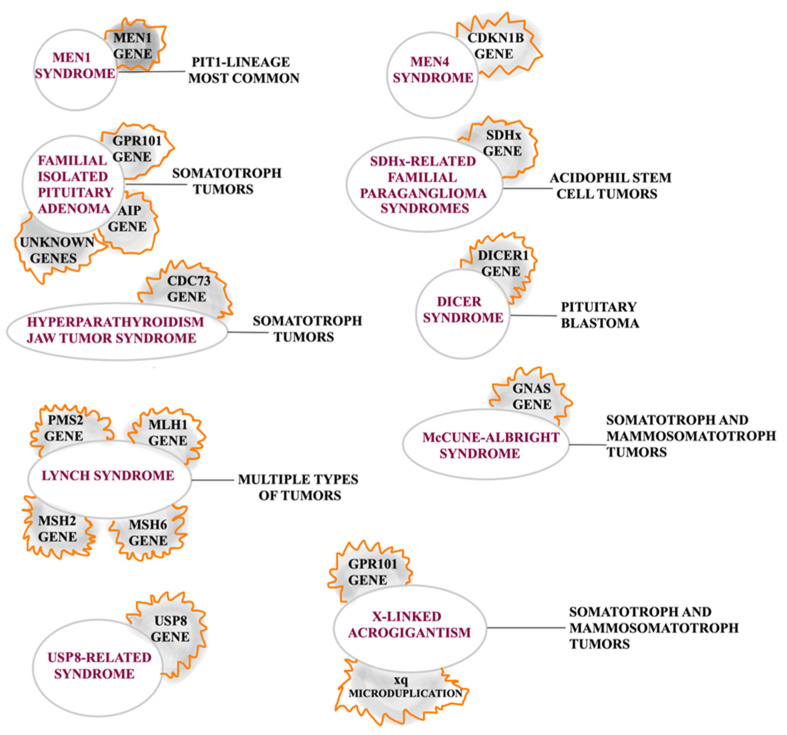
Summary of inherited pituitary tumors.

Approximately 5% of PAs arise in a hereditary context in patients with germline molecular alterations that incline toward the development of this pathology, usually resulting in tumors presenting at younger ages [23]. Other germline forms associated with these tumors are (1) neurofibromatosis type 1, with characteristics of acromegaly with an excess of GH and IGF1; (2) tuberous sclerosis, with genetic alterations of Tuberous Sclerosis Protein (TSC) 1 or TSC2 genes that were also found in patients with Pas; (3) germline CABLES1 types, as it has been found that the expression of the CABLES1 protein is lost in more than half of corticotrope adenomas (CAs); and (4) a CDH23 mutation which needs further investigations, as it is still unclear how it can influence the pituitary tumorigenesis [24]. It has also been considered that X-linked acrogigantism and familial isolated pituitary adenoma (FIPAs) are isolated presentations, while the rest are considered syndromic [25]. Although inherited pituitary tumors will be further discussed in the subsection of each PA subtype, a summary is presented in Figure 4.

### 2.2. Intracellular Signaling Pathways

In standard conditions, intracellular signaling pathways are the main factors responsible for cell survival, proliferation, differentiation, protein synthesis, and cellular metabolism. In PAs, these pathways are represented by PI3K/Akt/mTOR and Raf/Mek/ERK, which are upregulated and involved in the tumor’s genesis [26]. The PI3KCA gene is well known in PAs, as it has been stated that its amplification is linked to invasiveness and recurrence. In the PI3K/Akt/mTOR pathway, growth factors activate receptor tyrosine kinases (RTKs) that will start a signal transduction component (PI3K), which will further activate Akt, and it will finally lead to cellular proliferation [26]. Furthermore, a rise in Akt mRNA and B-Raf mRNA activity, expression, and phosphorylation has been noted in PAs. Additionally, in corticotrophinomas, a mutation of V600E BRAF was observed in as much as 16% of the tumors [27]. Regarding the Raf/Mek/ERK pathway, cellular proliferation results from multiple interconnections between different biomarkers. MEK1 and MEK2 are activated by Ras GTPase stimulation. GTPase stimulation is also responsible for Raf phosphorylation [28]. Subsequently, the activation and phosphorylation of ERK1 and ERK2 results in the modulation of many genes linked to proliferation [28].

It is worth mentioning that mTOR represents a significant downstream target of the PI3K/Akt/mTOR and Raf/Mek/ERK pathways [29]. Mutations in the mTOR, mTORC1, and mTORC2 pathways have recently been implicated in PAs, and new therapies based on their inhibition show promising results [30].

### 2.3. Epigenetics

The term “epigenetic” has developed to comprise any mechanism that alters gene activity without modifying the DNA sequence and conducts changes that can be passed on to daughter cells [31]. In PAs, epigenetic alterations majorly impact the availability of affected areas for RNA or DNA polymerases, which can influence tumoral development. The overexpression of DNA methyltransferase 1 and 3 (DNMT1 and 3) has been issued in macroadenomas. Thus, the suppression of these enzymes could turn out to have a successful antitumoral effect. Moreover, the higher expression of DNMT1 and 3 was correlated with aggressiveness and high-methylation status [32]. Other notable biomarkers with significant hypermethylation or hypermethylated promoters were the RB1 (tumor suppressor retinoblastoma protein), CDK1, CDKN1B, CDKN2A, CDKN2, and FGFR2 [33]. Missing transcript levels of Neuronatin (NNAT), a protein involved in the embryogenesis of the nervous system; Ras Association Domain Family Member 1 (RASSF1A); and the death-associated protein kinase (DAPK) have been found in more than 50% of PAs, which make them significant predictors of tumoral progression [34,35,36]. Although PAs are commonly benign, the invasive feature and aggressive behavior of these tumors may link them to a malignant progression, and epigenetic modifications in the methylation pattern may provide details about this type of tumoral conversion [37].

### 2.4. Somatotroph Adenomas

Somatotroph adenomas (SAs) comprise up to 20% of PAs, and by hypersecreting growth hormone (GH) they determine acromegaly, which leads to significant morbidity and mortality [38]. Currently, therapeutical management is based on the biochemical normalization of GH levels and the control of age-adjusted insulin-like growth factor 1 (IGF-1), surgical management, and radiotherapy as adjunctive to surgery [39]. One of the most cited predictor factors for treatment response is represented by the histological subtype, specifically densely granulated adenomas, and sparsely granulated adenomas. Thus, it has been stated that densely granulated adenomas tend to respond better to medical therapy, while sparsely granulated adenomas respond better to surgical treatment and are more resistant to medical therapy [38,40] while being considered aggressive tumors [41].

Generally, SAs have both somatic and germline mutations, which can cause elevated cyclic adenosine monophosphate (cAMP) levels, promoting cellular proliferation processes [42]. Somatic mutations have been mainly associated with the activation of the Guanine Nucleotide binding protein, Alpha Stimulating 1 (GNAS1), which supports not only tumoral proliferation but also the hypersecretion of GH in approximately 30% of SAs [43,44]. Moreover, somatic mutations in somatotropinomas, although scarce, have a major impact on calcium and ATP pathways, which are pivotal elements in PA tumoral development [45,46].

Germline mutations were associated with Aryl Hydrocarbon Receptor-Interacting Protein (AIP), G Protein-Coupled Receptor 101 (GPR101), Protein Kinase CAMP-Dependent Type I Regulatory Subunit Alpha (PRKAR1A), multiple endocrine neoplasia type 1 (MEN1), and cyclin-dependent kinase inhibitor 1B (CDKN1B) [47,48], as well as with the Succinate dehydrogenase gene (SDHx) and myc-associated factor X (MAX) [49].

Although PIT1 is the transcriptional factor for somatotroph adenomas [16], cases of pluripotent adenomas have also been described in the medical literature [50].

Although pituitary tumor-transforming gene (PTTG) overexpression has been noted in all PAs promoting DNA damage, the highest overexpression has been significantly correlated with SAs and GH secretion [51]. Consequently, in these tumors, the DNA damage and p53 induction of CDKp21 leads to cellular senescence. The fact that GH is not produced in cells where p53 is absent and the two bind each other while p53 increases GH secretion and transcription demonstrates that GH can be a p53 target and can be a biomarker in the senescence process mediated by p53 [52].

Even with all of the already-known information, the complete molecular processes behind the apparition of SAs still need to be fully understood and are still to be fully uncovered.

### 2.5. Lactotroph Adenomas

Lactotroph adenomas (LAs) account for up to 50% of all PAs and are described as being the most frequent functioning PitNET with a female predominance [53]. All three histological subtypes of LAs (sparsely granulated, densely granulated, and acidophil stem cell) have the same transcriptional factors, respectively PIT1 and ERα [54]. It has been stated that in post-menopausal women as well as in men, a combination of lower expression of ERα and estrogen hyposecretion supports tumoral cell growth while allowing the development of more aggressive tumors [55]. Furthermore, LAs in men are considered aggressive tumors [41] and sex-related differences have been discovered on a molecular level. Cancer/Testis Antigen 2 (CTAG2); vascular endothelial growth factor (VEGF), whose expression is higher in male patients; and fibroblast growth factor 13 (FGF13) were associated with the X chromosome while being part of the ER pathway, and ERα expression as well as FGF13 are correlated with tumoral aggressiveness and invasiveness in men [56].

Although prolactin hypersecretion is representative of these tumors, fugitive acromegaly has been described as a common feature of acidophil stem cell LAs. It usually reveals a voluminous tumoral mass [57] and secretes and expresses GH [58].

Other than the neurosurgical approach, these tumors can be treated with dopamine agonists (given their overexpression of dopamine D2 receptors), as well as with estrogen or estrogen–progestin combinations in women who do not want to recover fertility [59,60].

Approximately 3% of LAs appear in the context of familial syndrome [61], such as MEN 1 and 4, familial isolated pituitary adenomas (FIPAs), and Carney syndrome. Hereditary paraganglioma–pheochromocytoma syndrome with PAs is a very rare entity [62], and the most frequently cited cause is represented by alterations of Succinate dehydrogenase (SDH) subunits, such as A, B, C, D, and AF2 [63]. Outside familial settings, LAs are usually sporadic; however, tumors with a genetic cause of apparition have been described in association with germline mutations such as Endoribonuclease Dicer (DICER1) [64]. It is worth mentioning that somatic mutations are rare in these tumors, while germline mutations are described in both familial and sporadic contexts. Mutations regarding the AIP gene have been discovered in up to 31% of FIPAs [65], while only 2.6% of patients with PAs, including LAs, harbor this mutation [66].

Thrombospondin motif 6 (ADAMTS6), Collapsin Response Mediator Protein 1 (CRMP1), Pituitary Tumor-Transforming Gene 1 Protein (PTTG), apoptosis signal-regulated kinase (ASK), Cyclin B1 (CCNB1), Aurora Kinase B (AURKB), and Centromere Protein E (CENPE) genes were associated with tumoral relapse and the progression of LAs [67].

The transcriptomic analysis concluded that miRNA deregulation has a pivotal role in LAs. Thus, a decrease in miR-23b and miR-98 expression was correlated with aggressiveness, while miR-183 impacts the expression of genes at the mRNA and protein levels, promoting aggressiveness [68].

The allelic loss of chromosome 11 has been shown to be associated with tumor aggressiveness as a consequence of deregulating many genes located in this area, such as the CD44 molecule (CD44), Tumor Susceptibility 101 (TSG101), Diacylglycerol Kinase Zeta (DGKZ), HIV-1 Tat Interactive Protein 2 (HTATIP2), and General Transcription Factor IIH Subunit 1 (GTF2H1) [69].

The dysregulation of CRB3, FAM138F, MATK, and STAP2 genes located on gained regions of chromosome 19 was also associated with aggressive LAs [56]. In contrast, chromosome 12 abnormalities with the High-Mobility Group A2 gene (HMGA2) rearrangements and higher expression have been described [70].

### 2.6. Corticotroph Adenomas

Corticotroph adenomas (CAs) represent approximately 15% of all PAs [71], and they appear more frequently as microadenomas, especially in young women [72]. Notwithstanding the vast majority of these tumors secreting ACTH and causing Cushing’s disease, a minority of up to 10% are considered silent, as they stain positively for ACTH despite no higher levels of cortisol being recorded [73]. Furthermore, silent CAs are considered more invasive, with higher recurrence rates. Although these tumors have very high proopiomelanocortin (POMC) levels, the prohormone convertase 1/3 (PC1/3) that converts it into ACTH is deficient; however, type II silent CAs are more prone to full conversion recovery, which may suggest a thorough follow-up [73].

Histologically, CAs were classified as densely granulated (DGCA), sparsely granulated (SGCA), and Crooke cell adenoma, and in all cases, the transcriptional factor is represented by TPIT [74]. Crooke cell adenomas are usually more voluminous, are invasive into the surrounding anatomical structures, and exhibit aggressive behavior, sometimes even with a transformation into metastatic pituitary carcinoma [75].

In approximately 60% of cases, Ubiquitin-specific peptidase 8 (USP8) is the responsible genetic cause of CA apparition [75]. Furthermore, USP8 is associated with cell proliferation and invasiveness through links with the epidermal growth factor receptor (EGFR) [76], as well as Nuclear Receptor Subfamily 3 Group C Member 1 (NR3C1) [77], B-Raf Proto-Oncogene, Serine/Threonine Kinase (BRAF), and Ubiquitin-specific peptidase 48 (USP48) [78]. The EGFR is overexpressed in up to 55% of CAs and positively correlates with hormonal levels and tumoral recurrence [76]. Mutations involving Cdk5 and Abl Enzyme Substrate 1 (CABLES1), MEN1, PRKAR1A, CDKN1B, USP8 variants and AIP genes were described as sporadic in pediatric patients [79]. Clinical behavior, cell proliferation, and invasiveness were related to testicular orphan nuclear receptor 4 (TLR4) [80], heat-shock protein 90 (HSP90) [81], and the miR-106b-25 cluster [82]. Additionally, Yamamoto et al. describe BRAF, CABLES1, DICER1, Tumor Protein P53 (TP53), and Alpha Thalassemia/Mental Retardation Syndrome X-Linked (ATRX) as genes responsible for Cushing’s disease that are correlated with aggressive behavior [75]. Sbiera et al. demonstrated a concurrent mutation in both ATRX and TP53 [83], while Borota et al. concluded that loss of ATRX is not only more common in CAs but can also be associated with aggressiveness [84].

### 2.7. Thyrotroph Adenomas

Representing less than 1% of all PAs, these tumors are not linked to either germline or somatic mutations, and they are characterized by their thyrotropin-secreting feature [85]. However, plurihormonal cases were reported [86,87]. Thyrotroph adenomas (TAs) are different from other PAs in a genetic context as they do not have a hereditary background. However, rare cases of association with MEN1 were described and correlated with loss of heterozygosity on 11q13. However, the results showed a wild-type gene, which concluded that the MEN1 gene is not involved in the molecular pathogenesis of TAs [88].

In these tumors, the transcriptional factors are represented by PIT1 and GATA3, while the hormones by immunohistochemistry are represented by β-TSH and the α-subunit [53]. It has been demonstrated that PIT1 was overexpressed in TAs while having a pivotal role in cellular proliferation [87].

The general molecular processes behind TAs’ development are yet to be fully understood. In contrast with other PAs, no oncogenic mutations were observed in these tumors when screened for alpha q, s, and 11 and TRHR genes, but others are yet to be studied [89]. Rare cases of AIP mutations in TAs were described in the medical literature [90,91]; however, chromosomal alterations were most frequently present in these tumors compared to in other PAs [92].

It has been stated that the 135 bp deletion is caused by the aberrant binding of mRNA and TRβ2, which results in an atypical TR protein. These findings may explain why thyroid hormones as inhibitory treatments do not produce results [93].

### 2.8. Gonadotroph Adenomas

Clinically functioning gonadotroph adenomas (GAs) represent approximately 35% of all GAs, expressing and secreting the FSH and LH, leading to hypersecretion syndrome; however, the majority of GAs are hormonally silent and characterized by tumoral mass effect [94]. The main therapeutic option in functional GAs is tumoral resection, given that they do not respond very well to medical conservative treatments [95]. Transcriptional factors are represented by SF1, GATA3, and ERα [42].

Cell proliferation has been connected to the noncoding RNA Maternally Expressed Gene (MEG), which regulates p53 gene expression [96].

Given their scarcity, the exact molecular mechanisms behind the apparition of clinically functioning GAs are still under research. It has been stated that the expression of the GnRH gene is more frequent in these tumors than in nonfunctioning ones, and no regional mutations were discovered, leading to the conclusion that this mechanism is not involved [97].

### 2.9. Null Cell Adenomas

Null cell adenomas represent approximately 0.6% of all PAs and are characterized by no lineage differentiation, transcriptional factors, or hormone secretion. They are also usually larger in volume than other PAs [98,99].

Specific mutations were associated in these tumors with cell proliferation, invasion, tumor development, and apoptosis resistance. These mutations were identified in Platelet-Derived Growth Factor D (PDGF-D), sterile alpha motif and leucine zipper-containing kinase (ZAK), and Phosphatidylinositol-4,5-Bisphosphate 3-Kinase Catalytic Subunit Alpha (PIK3CA) [100]. Moreover, epigenetic changes involving Growth Arrest and DNA-Damage-Inducible Alpha (GADD45) were correlated with tumoral growth and evolution [101].

While hypermethylation of cyclin-dependent kinase inhibitor 2A/multiple tumor suppressor gene 1 (CDKN2A/MTS1/p16) was correlated with an early stage of tumoral development [102], microRNAs that target Smad proteins and C5orf66-AS1 were associated with tumoral size in null cell PAs [103].

### 2.10. Landscape of Molecular Events in Pituitary Tumor Apoplexy

Pituitary tumor apoplexy is defined by intratumoral hemorrhage and/or infarction. An early diagnosis may be crucial, as it can be a life-threatening condition, and prompt medical and surgical treatment may be essential [104]. Although, in this case, the molecular pathways in their entirety are not yet clarified, it is well known that a small vascularization of PAs is consequential, as it triggers various biomolecular mediators responsible for tumoral hemorrhage [104].

Multiple genetic factors are involved in PAs, each with a specific role. Thus, the vascular endothelial growth factor (VEGF) has a pivotal role in tumor angiogenesis, Endolin (CD105, CD31) has a role in microvascular density, the pituitary tumor-transforming gene (PTTG) and fibroblast growth factor (FGF) are involved in pituitary tumorigenesis and development, while the Ki67 proliferation marker has a role in cell proliferation [105].

Another important element in hemorrhagic events of apoplexy is represented by tumor necrosis factor-alpha (TNF-α), which is involved in angiogenesis, vascular hyperpermeability, and the destruction of vascular integrity [106]. In addition, HIF-1α (hypoxia-inducible factor 1-alpha) is involved in hypoxia and the activation of the VEGF [107,108], while matrix metalloproteinase-2/9 (MMP-2/9) is responsible for the degradation of extracellular matrix and vascular permeability [109,110]. HIF-1α in PAs increases the risk of pituitary apoplexy by stimulating the expression and synthesis of the VEGF (which influences MAPK, FAK, PI3K/Akt, p38 MAP kinase signaling pathways), MMP 2, TGF-β, and the signaling pathway Wnt [107,108]. Pituitary apoplexy is more common in nonfunctioning macroadenomas (showing elevated microRNA values for the VEGF) than in pituitary microadenomas [111]. However, further research is needed to gain more knowledge about the biomolecular pathways that lead to pituitary apoplexy so that better management of this condition can be acquired.

## 3. Conclusions

The multifactorial fundamental processes regarding pituitary adenoma’s molecular pathology require a variety of interactions between modern multi-omics. The development of these tumors involves mechanisms concerning genetic or epigenetic changes, which may be critical in obtaining a reliable and detailed diagnosis.

While inherited PAs tend to have a clearer biomolecular background, with isolated or syndromic presentations, non-inherited PAs are still being discovered, as the advancement of molecular biology has begun to shed light on the genetic mutations behind the development and evolution of this pathology. Currently, these tumors are categorized based on transcription factors and hormone production and they generally have both somatic and germline mutations.

As studies are being conducted on the favorable influence of the epigenetic elements involved in the etiopathogenesis of these tumors, future efforts will be needed to ensure precise improvements regarding the molecular landscape and to further our understanding of the molecular biology of each PA subtype.

## Figures and Tables

**Table 1:** Summary of the main differences and similarities between aggressive and refractory PAs.

	Aggressive PAs	Refractory PAs
**Resistance to standard therapies**	Yes	Yes
**Cerebral/spinal/distant metastasis**	No	No
**Tumor invasion**	Proven on neuroimaging results	Proven intraoperatively and on neuroimaging results
**Growth velocity**	Not necessarily required	>2%/month
**Recurrence**	Not necessarily required	In less than 6 months after surgical removal
**Ki67 index**	Not necessarily required	>3%

## Data Availability

No new data were created or analyzed in this study.
